# A review of the pattern of AIDS defining, HIV associated neoplasms and premalignant lesions diagnosed from 2000–2011 at Kenyatta National Hospital, Kenya

**DOI:** 10.1186/s13027-015-0021-1

**Published:** 2015-08-24

**Authors:** Emily A. Rogena, Kenneth O. Simbiri, G. De Falco, L. Leoncini, Leona Ayers, J. Nyagol

**Affiliations:** Thematic Unit of Anatomic pathology, Department of Human pathology, College of Health Sciences, University of Nairobi (KNH CAMPUS), PO BOX 55050 00200, Nairobi, Kenya; Department of Microbiology and Immunology, SUNY Upstate Medical University, New York, USA; School of Biological and Chemical Sciences Queen Mary University of London, London, UK; Department of Medical Biotechnologies, University of Siena, via Delle Scotte, 6, Siena, 53100 Italy; Mid region AIDS Cancer specimen resource, (NCI), Ohio State University, 2046, Innovation Centre 2001 Polaris Parkway, Columbus, Ohio 43240 USA; Thematic Unit of Immunology Department of Human pathology, College of Health Sciences, University of Nairobi (KNH CAMPUS), PO BOX 19676 00202, Nairobi, Kenya

## Abstract

**Background:**

Sub-Sahara Africa hosts up to 71 % of all HIV infected people in the world. With this high incidence of Human immunodeficiency virus ( HIV) comes the burden of co-morbidities such as malignant and premalignant lesions. Aids defining malignancies have been listed as Kaposi’s sarcoma, Non-Hodgkin’s lymphoma and invasive squamous cell carcinoma of the cervix. People with HIV/AIDS(PLWAS) have a higher risk of developing these neoplasms than the rest of the population. The pathogenesis of these neoplasms in people with HIV has been linked to immune suppression, persistent antigenic stimulation and cytokine dysregulation.

Current study analyzes and presents the patterns and trends in the presentation of HIV related malignancies in patients diagnosed through histopathology at Kenyatta National Hospital.

**Aim:**

To describe the patterns of AIDS- defining and non-AIDS- defining malignancies and premalignant lesions 10 years pre- and post HAART period at Kenyatta National hospital, Kenya.

**Methods and techniques:**

This was a hospital based descriptive cross sectional study. The Formalin fixed paraffin embedded (FFPE) blocks and histological reports of patients diagnosed between 2000 and 2011 were traced from archives. The patients’ demographic data and clinical presentation was entered in an excel spreadsheet and the diagnosis and coding confirmed by a histopathologist. The data was then cleaned and analyzed using SSPS version 17.0 Ink.

**Results:**

A total of 173 lesions were reviewed and analyzed. Of these 118 (68 %) were from females and 55 from males (32 %). The male to female ratio was 1:2. The age range was from two to 56 years with a median of 36 years. Kaposi sarcoma is the leading AIDS defining malignancy in Kenya while invasive squamous cell carcinoma of the conjunctiva is the leading non-AIDS defining malignancy. This is closely followed by invasive squamous cell carcinoma of the cervix and NHL.

**Conclusion:**

Kaposi sarcoma is the leading AIDS associated neoplasm in Kenya. Physicians and caretakers managing and following up on HIV/AIDS patients should look out for Kaposi sarcoma as a form of IRIS following the institution of HAART in all HIV/AIDS patients. The incidence of invasive squamous cell carcinoma of the conjunctiva is increasing in PLWAS in Kenya. There is therefore a need to introduce early screening programs for squamous intraepithelial neoplasm of the conjunctiva in HIV/AIDS patients.

## Introduction and background

Sub-Saharan Africa hosts up to 71 % of all adults and children living with HIV, 70 % of adults and children newly infected, and 75 % of adults and children dying from AIDS [1]. In Kenya 1.6million people were living with HIV, with a prevalence rate of 6.1 % in adults 15–49 years of age, and death due to AIDS of 57,000 in 2012 [1]. With this high incidence and prevalence of HIV comes the burden of co-morbidities such as malignant and premalignant lesions [2]. AIDS defining malignancies have been listed as Kaposi sarcoma, Non-Hodgkin’s lymphoma and invasive squamous cell carcinoma of the cervix [3, 4]. People with HIV/AIDS have a higher risk of developing these neoplasms than the normal population [3]. The pathogenic mechanisms of these neoplasms are still not well understood although several authors suggest immune suppression, persistent antigenic stimulation, and elevated pro-inflammatory cytokines and other viruses as cofactors. [2]. Some of the cytokines that have been associated with these cancers include vascular endothelial growth fact VEGF, Kaposi’s sarcoma growth factor KSGF, IL-6, and HIV Tat protein and IFgamma and alpha [5, 6]. These cytokines and factors influence the cell cycle and hence proliferation via several mechanisms that will not be discussed in this article. Although the sub-Saharan Africa leads in the incidence of HIV/AIDS, data on trends of AIDS associated malignancies in African countries is scanty [7–9]. This is partly attributed to the lack of well-established cancer registries [7–11], as is the case in Kenya. Most of the data in literature emanates from Uganda, possibly due to the presence of an active Kampala cancer registry which has been in existence since 1954 [12]. Literature from the developed countries show that following the introduction of HAART, the incidence of KS was significantly reduced amongst HIV/AIDS patients [2]. In addition the incidence of other neoplasms that are not AIDS defining have increased in these patients [2]. There has been an increase in the incidence of other neoplasms also referred to as non-AIDS defining neoplasm such as Hodgkin’s lymphoma, myeloma, hepatocellular carcinoma and gastric adenocarcinoma [2]. It is against this background that we set out to review the trend of HIV related malignancies seen at our department of Pathology between 2000 to 2011. In this study we describe the patterns of AIDS- defining and non-AIDS- defining malignancies and premalignant lesions 11 years pre- and post HAART period at Kenyatta National hospital Kenya (Table 1).

**Table 1:** Distribution of the types of neoplasms and premalignant lesions by site

SITE OF BIOPSY	HL	Kaposi sarcoma	Squamous cell carcinoma	Adenocarcinoma	Burkitt lymphoma	Aggressive NHL	Carcinoma-in-situ/severe dysplasia	Ductal carcinoma	Hepatocellular carcinoma	High grade sarcoma	Total
Skin		35	1	0	0	2	0	0	0	0	38
Cervix/Vulva	0	0	10	0	0	0	20	0	0	0	30
Orbit/Eye/Conjunctiva	0	3	22	0	0	0	8	0	0	0	33
Appendix	1	15	1	0	1	5	0	0	0	1	24
Stomach/Gastric	0	0	1	1	0	0	0	0	0	0	2
Uterus	0	0	1	0	0	0	5	0	0	0	6
Breast	0	0	0	0	0	0	0	1	0	0	1
Bone/Soft tissue	0	0	2	1	0	0	0	0	0	0	3
Tongue/Oral cavity/gingival	0	14	5	0	0	0	0	0	0	0	19
Oropharynx/palate/PNS nose	0	15	0	0	0	0	0	0	0	0	15
Liver	0	0	0	0	0	0	0	0	1	0	1
Penis	0	0	1	0	0	0	0	0	0	0	1
Total (4 sites were not recorded)	1	82	173	173	173	173	173	173	173	173	173

## Methods and techniques

Having obtained approval from the KNH/UON ethics and research committee. The data clerk at the AIDS and Cancer Specimen Resource-Kenya (ACSR K) retrieved all the reports indicated to be HIV positive on the histological request forms between 2000 and 2011. This was done by checking for clinical information on the form indicating p24 marker positive (p24 is a marker of HIV antigenicity in serum), retroviral disease, if patient was on ARV or HAART treatment, or patient on follow-up at the comprehensive care clinic (CCC). Using the histological reports, blocks for the respective cases were then retrieved from the routine block storage and archived. 4 The criteria for inclusion as HIV positive case included information from the clinician in the patient’s histological request form indicating follow up at the CCC, HAART treatment, ARV treatment, immunosuppression, p24 marker reactive, retrovirus disease, and HIV positive.

The patients’ demographic and clinical data from the histological request form and partly from the hospital medical records were entered into Microsoft Excel 2010. Ink spreadsheet. The data was cleaned and analyzed using SPSS statistics 17.0 Ink.

## Results

**Fig. 1:**
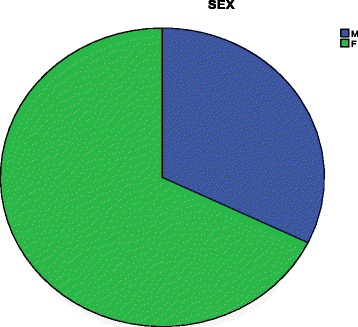
Distribution of the cases by sex. The Pie chart shows the distribution of males and females in the study. There were 68.2 % female and 32.8 % male

**Fig. 2:**
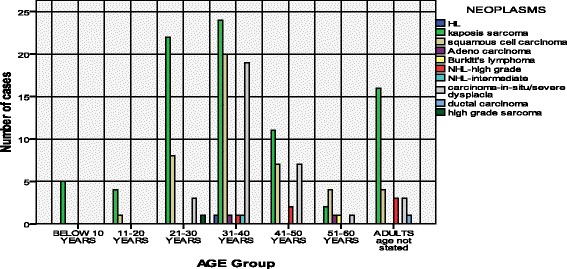
The distribution of types of Lesions by age. The figure shows the distribution of cancers by age of diagnosis in HIV-1 cases in Kenyatta National Hospital between 2001 and 2011

**Fig. 3:**
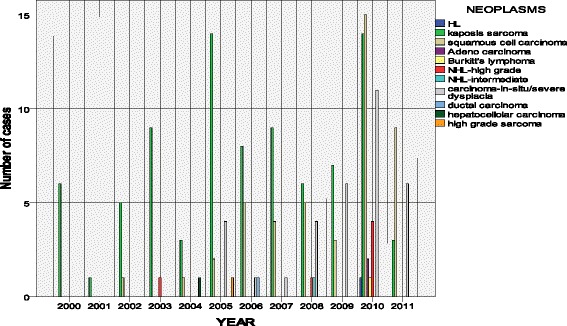
The distribution of neoplasms Reported between 2000 and 2011. The figure shows the cancers identified in Kenyatta National Hospital in 2001–2011 using patient charts and FFPE

**Fig. 4:**
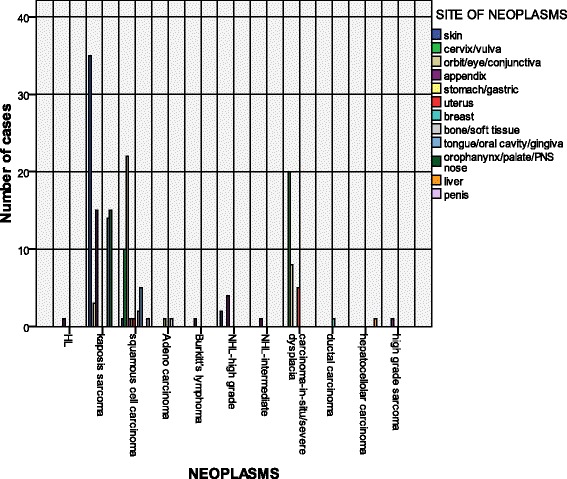
The distribution of neoplasms by site. The figure shows specific types of neoplasms detected at Kenyatta National Hospital with respect to site

A total of 173 lesions were reviewed and analyzed by the study histopathologists (Ndungu, JR, Rogena, EA). Of these 118 (68 %) were females and 55 males (32 %). The male to female ratio was 1:2. The age range was from 2 to 56 years with a median of 36 years (Fig. 1). Between the year 2000 and 2003, the main neoplasm reported was Kaposi’s sarcoma and squamous cell carcinoma. Starting from 2003, other neoplasms such as High grade Non-Hodgkin’s lymphoma and gastric adenocarcinoma began to emerge. Kaposi’s sarcoma represents the largest number of cases at 48 % followed by squamous cell carcinoma of the conjunctiva and cervix/vulva at 12.5 % and 5.6 % respectively. Aggressive Non-Hodgkin’s lymphoma account for 4.6 %; of these lymphomas and Burkitt lymphoma make up 0.6 %. The premalignant lesion, carcinoma-in-situ (CIS),is seen both in the cervix and the conjunctiva. However, squamous cell carcinoma is more prevalent in the conjunctiva (Fig. 2). In our sample the aggressive non-Hodgkin’s lymphomas are found in the less common sites such as the appendix and the skin. The only Burkitt lymphoma reported was in an adult, involving the appendix. Kaposi’s sarcoma is also seen frequently in the appendix and the oropharynx. Of interest is the lack of ano-rectal carcinoma in this series (Figs. 3 and 4).

## Discussion

The male to female ratio of 1:2 reported to have AIDS Related malignancies is in keeping with the higher incidence of HIV infection in the female population in Kenya as shown by the National HIV indicators for Kenya 2010 (National HIV Indicators for Kenya: 2010 National AIDS Control Council and the National AIDS and STD Control Program). The female gender is at a higher risk of acquiring HIV infection due to many factors such as the anatomy and the immunity of the reproductive system and socio-economic factors [1, 11]. Over the years Kaposi sarcoma, squamous cell carcinoma of the uterine cervix and High Grade Non-Hodgkin’s lymphomas have been listed as AIDS defining malignancies. The AIDS defining malignancies are neoplasms that consistently correlate with the presence of AIDS in HIV infected persons. In a pilot study by Korir et al. in Kenya, the authors observed that of 171 cases, 118 had tumors associated with HIV, including Kaposi’s sarcoma, cervical cancer, non- Hodgkin’s lymphoma, Hodgkin’s disease, and conjunctival carcinoma [11]. The non-AIDS defining malignancies appear to occur at much younger age in HIV infected persons compared to those that are HIV negative, the neoplasms show atypical features, and a higher grade and stage at the time of diagnosis [12].

The data in this study reveals a progressive increase in the number and variety of malignancies and premalignant lesions diagnosed since the introduction of HAART in 2003. The trend is such that Kaposi’s sarcoma and squamous cell carcinoma of the cervix and vulva predominated between 2000 and 2003, the pre HAART period in Kenya. Kaposi’s sarcoma is still leading, but incidence of squamous cell carcinoma of the conjunctiva is surpassing that of the cervix and vulva. Later in 2003, majority of HIV patients started receiving ARV from MEDS (Mission for essential drug suppliers- a catholic church NGO). Following this, in the same year, the Kenya government announced the inception of HAART treatment through public hospitals which were implemented through funding by GLOBAL and PEPFAR funds in 2004 [15]. Over the period of eight years, up to 1million Kenyans have been put on HAART. One expects an increase in the survival of people living with HIV/AIDS. The developed countries over the years have recorded a significant decrease in the incidence of AIDS related malignancies since the introduction of HAART in those settings [16]. In Kenya, the trend appears to demonstrate a relative increase in the number of cases and the variety of AIDS related malignancies. This could be accounted for by the survival rate of these patients as the HAART programs become widespread. Another factor that may contribute to the high incidence is the endemicity of KSHV (HHV8) in East Africa. In this study we did not actively review the information on the correlation of HAART use and the neoplasm. Nor did we correlate the KSHV marker, LANA 1 marker and the presence of Kaposi’s sarcoma as objectives of the study.

Reports from Kampala, Uganda show that HIV related Kaposi’s sarcoma affects mainly the younger and middle age groups, and the median age in 1990s being 32.0 years and 27.1 years for men and women respectively. This distribution is similar to the median age of AIDS cases recorded for the country in 1996 of 34 years and 30 years in men and women respectively [12]. KS can involve any site in HIV infected persons, including bone marrow, pancreas, gastrointestinal tract (GIT), lungs, liver, heart, and testis [17]. The oral cavity, palate and larynx are other common sites. Of the 82 cases of Kaposi’s sarcoma reported in this study (42.7 %) showed involvement of KS in the skin, the appendix and oro/nasopharynx/palate represented 15 % each, and the conjunctiva and tongue/oral mucosa was at 3.7 % and 17.1 % respectively. Kaposi’s Sarcoma associated with HIV is said to be more aggressive and has been shown to occur at these unusual sites [12]. This observation has led to the proposal that HIV-1 encoded proteins such as Tat protein may induce some cytokines that work synergistically with products of HHV8 [16, 18, 19]. Tat interacts with RB2/P130 tumor suppressor gene among other pathways [20]. KS may flare up upon introduction of HAART in some cases. This is thought to be due to Immune reconstitution syndrome, a reaction to some of the infections seen in HIV [21]. Due to the scanty nature of the clinical information accompanying histological specimens to the laboratory, one would not easily elucidate this syndrome using laboratory data. Recent studies from Kenya, Uganda and South Africa show that HAART is effective in reducing the standardized incidence of KS [22].

Invasive squamous cell carcinoma of the cervix is classified as one of AIDS defining malignancies. The occurrence of this neoplasm in HIV represents a progression to AIDS. The neoplasm is directly linked to HPV, a virus that has been shown to transform squamous cells through the E6 and E7 proteins. These proteins act via the cyclin dependent kinases E/CDK2 to phosphorylate and inactivate RB gene. Proposals have been put forth suggesting that HIV may upregulate E6 and E7 through Tat [23]. Whereas the pathogenesis of squamous cell carcinoma of the conjunctiva and that of the cervix is similar, the study shows that invasive squamous cell carcinoma of the conjunctiva has surpassed that of the uterine cervix. On the other hand CIS is more prevalent in the uterine cervix than in the conjunctiva. This reversed trend could be as result of the cervical screening programs in Kenya, targeting all women. However, there is no such program targeting the conjunctiva in PLWAs in Kenya. A review of HIV related cancers in Uganda revealed a dramatic increase in invasive squamous cell carcinoma of the conjunctiva from four cases reported between1960 to 1971 and 66 cases reported between 1994 and 1997 [12]. Similarly, Ateenyi from Uganda demonstrated similar positive correlation between squamous cell carcinoma of the conjunctiva and HIV in 1995 [24]. Although cancer of the cervix is classified as AIDS defining, Uganda series do not show a significant risk between HIV positive and HIV negative women. However, the Ugandan reports reveal a significant link between CIS and HIV infection [16]. Nonetheless, lesions diagnosed through screening programs have to be interpreted with caution taking into consideration the biases introduced by screening programs that lead to the diagnosis of these premalignant lesions such as CIS.

In general aggressive B cell lymphomas are one of the nightmares in management of HIV/AIDS. These neoplasms are usually non-responsive to routine protocols. Diffuse large B cell lymphomas form the largest percentage followed by Burkitt lymphoma. Non Hodgkin’s lymphoma put together accounted for 4.6 % of all the neoplasms and premalignant lesions in this study. In the United States, the relative risk of NHL in subjects with AIDS is 100 or more and much higher than the relative risk in an African series reporting that shows Rwanda leads at 13 [95 % confidence interval (CI), 2.2–44.4] [25] and South Africa at 4.8 (95 % CI, 1.5–14.8) [26]. The risk of developing lymphoma steadily increases with duration of HIV infection and advancing immunosuppression. Prospects for increased survival are enhanced by HAART; however, long-term survivors of HIV infection may remain at increased risk for NHL [27]. The slow roll out of HAART use may partly account for death of HIV/AIDS patients earlier in this setting prior to the diagnosis of lymphomas hence the low reported incidence in this study.

Burkitt lymphoma in sub-Saharan Africa is mainly a childhood disease accounting for up to 82 % of all pediatric and adolescent lymphomas in the region [28]. HIV related BL however occurs mainly in adults as shown in this study and presents at unusual sites. All the cases of Burkitt lymphomas in the HIV/AIDS patients were reported between the ages of 51 to 60 years. The numbers given in this study may be an underestimation since the main diagnostic method for Burkitt lymphoma has been Fine needle aspiration Cytology (FNAC). Therefore histological records would understate the incidence.

Recent data reported from several hospitals in East Africa reveal that Hodgkin’s Lymphoma is a disease of children and adolescents [28], the only case of Hodgkin’s lymphoma in this review was reported between the age of 31 to 40 years. The disease has been reported to be on the increase in HIV/AIDS [16]. There appears to be a linkage between EBV and HIV in the pathogenesis of lymphomas, a fact that calls for further research. Nearly all HLs are associated with EBV [23]. The EBV related proteins EBNA 1, EBNA 2, EBNA-LP, EBNA 3A and EBNA-3C and LMP differentially drive the cells through notch pathway, cell cycle check point (G2/M) and by activation of NF-κB [29]. We would like to point out that a number of the cases diagnosed in the period may not have been retrieved due to challenges in record keeping.

Although Burkitt lymphoma is one of the well reported high grade lymphomas seen in HIV/AIDS, the cases reported in this study were very few. Of note is that during the period under review, the main diagnostic method for Burkitt lymphoma was largely through FNAC, and may account for the low incidence of archived tissue of BL in this series.

## Conclusion

Kaposi sarcoma is the leading AIDS associated neoplasm in Kenya. The incidence of invasive squamous cell carcinoma of the conjunctiva is increasing in PLWAS in Kenya. There is therefore a need to introduce early screening programs for squamous intraepithelial neoplasm of the conjunctiva in HIV/AIDS patients.
